# Synergistic effects of depression and *NR3C1* methylation on prognosis of acute coronary syndrome

**DOI:** 10.1038/s41598-020-62449-2

**Published:** 2020-03-26

**Authors:** Hee-Ju Kang, Robert Stewart, Ju-Wan Kim, Sung-Wan Kim, Il-Seon Shin, Min-Chul Kim, Young Joon Hong, Youngkeun Ahn, Myung-Geun Shin, Myung Ho Jeong, Jin-Sang Yoon, Jae-Min Kim

**Affiliations:** 1 Department of Psychiatry, Chonnam National University Medical School, Gwangju, Korea; 2 King’s College London, Institute of Psychiatry, Psychology and Neuroscience, London, United Kingdom; 3 South London and Maudsley NHS Foundation Trust, London, United Kingdom; 4 Department of Cardiology, Chonnam National University Medical School, Gwangju, Korea; 5 Department of Laboratory Medicine, Chonnam National University Medical School, Gwangju, Korea

**Keywords:** DNA methylation, Predictive markers

## Abstract

High levels of methylation in the GR gene (nuclear receptor subfamily 3, group C, member 1; *NR3C1*) have been associated with depression and cardiovascular risk. This study aimed to investigate whether *NR3C1* methylation status was associated with the long-term prognosis of acute coronary syndrome (ACS) considering depression and cardiovascular status at the early phase of ACS. A total of 969 patients with recent ACS were recruited at a tertiary university hospital in Korea. Baseline evaluations were made from 2007 to 2012, including DSM-IV depressive disorder, *NR3C1* methylation, and various demographic and clinical characteristics such as cardiovascular risk markers. Over a 5~12 year follow-up after the index ACS, time to major adverse cardiac event (MACE) was investigated using Cox regression models. Higher *NR3C1* methylation status was associated with depression and several cardiovascular risk markers at baseline. *NR3C1* hypermethylation predicted worse long-term prognosis of ACS only in the presence of depressive disorder with significant synergistic interaction terms and independent of potential confounding factors. Synergistic effects of depressive disorder and *NR3C1* hypermethylation on long-term cardiac outcomes in ACS were found. *NR3C1* methylation status represents a candidate prognostic biomarker for ACS in combination with a diagnosis of depressive disorder. Further research is needed to ascertain the generalisability of these findings.

## Introduction

Depression is common in patients with acute coronary syndrome (ACS; including myocardial infarction (MI) and unstable angina (UA)), and is associated with poor prognosis including increased morbidity and mortality^1^. Common biological mechanisms underlying ACS and depression may account for this^2^. Dysregulation of hypothalamic–pituitary–adrenal (HPA) axis is one candidate, since this is strongly implicated in the pathogenesis of depression^3^ and also associated with impaired repair after cardiac injury^4^. The effects of cortisol, the major endogenous glucocorticoid hormone in the HPA axis, are principally mediated by glucocorticoid receptors (GRs), whose expression and sensitivity are modulated by epigenetic modifications^5^. DNA methylation, one of the widely investigated epigenetic modifications, refers to covalent methylation of the C5 position of cytosine residues followed by guanine residues (CpG dinucleotides)^6^.

High levels of methylation in the GR gene (nuclear receptor subfamily 3, group C, member 1; *NR3C1*) have been reported to be correlated with reduced GR expression in animals^7^, and in humans with depression^8^, post-traumatic stress disorder^9^, and suicidality^10^. With respect to ACS, hypermethylation of *NR3C1* has been found to be associated with atherosclerosis and elevated cardiovascular reactivity^11,12^. Recently, our study group found that higher DNA methylation of *NR3C1* was independently associated with depressive disorder at the early phase of ACS^13^. Based on these findings, it can be postulated that *NR3C1* methylation status may be associated with cardiac prognosis of ACS independently of or interactively with depression. However, the role of *NR3C1* methylation on prognosis of ACS has not been investigated, despite the clinical importance of depression-ACS comorbidity.

In the present study, we aimed to investigate whether *NR3C1* methylation status was correlated with known cardiovascular markers at the early phase of ACS, and whether it was associated with the long-term prognosis of ACS considering the negative impact of depression on these outcomes.

## Materials and Methods

### Study outline and participants

The analyses were performed using data from a large naturalistic cohort study which was set up to investigate the inter-relationships between depression and ACS: the DEPression in ACS (DEPACS) study. The design and main findings have been published^14,15^. The outline of the recruitment process for the present analysis is presented in online Fig. S1. ACS patients (N = 4809), recently hospitalized at the Department of Cardiology of Chonnam National University Hospital, Gwangju, South Korea, were approached consecutively to participate in the study from 2006 to 2012. With authorization by the Korean Circulation Society, this department has played the role of the central coordinating centre for the Korea Acute Myocardial Infarction Registry (KAMIR)^16^.

KAMIR is a web-based registry platform (http://kamir5.kamir.or.kr/) to collect nationwide multicentre data regarding clinical practices and outcomes of patients with acute MI prospectively which enables it to evaluate the prospective associations of a range of exposures or interventions with long-term cardiac outcomes. Treatment of ACS patients was based on international guidelines for the management of ACS^17^ by cardiologists participating in the present study. Those who met eligibility criteria (detailed in online supplementary material) and agreed to participate (N = 1152) received baseline evaluations as inpatients within 2 weeks (mean 6.3 ± SD 2.4 days) post-ACS. Of these, 969 (84.1%) agreed to phlebotomy and comprised the study sample. All participants were approached for follow-up evaluations for cardiac outcomes at 2017, 5~12 years after the index ACS. Written informed consent was obtained, and the study was conducted in compliance with institutional guidelines and the 1964 Declaration of Helsinki. Moreover, the present study was approved by the Chonnam National University Hospital (CNUH) Institutional Review Board.

### Baseline evaluation

Depressive disorders were diagnosed by psychiatrists with the Mini-International Neuropsychiatric Interview (MINI)^18^, a structured diagnostic interview for psychiatric disorders, according to criteria of the Diagnostic and Statistical Manual of Mental Disorders, Fourth Edition (DSM-IV)^19^, for defining major or minor depressive disorders.

For *NR3C1* methylation status, DNA was extracted from venous blood using standard procedures. The *NR3C1* region chosen for methylation analysis was exon 1 F (GeneBank #AY 436590), which includes a binding site for nerve growth factor-inducible protein A (NGFI-A) and is highly expressed in the hippocampus of pups with nurturing mothers^20^. Methylation status was measured in three CpG sites (Supplementary Fig. 2), located in the CpG-rich region of NR3C1 exon 1 F between −3166 and −3147 (CGGTGGCCCTCTTAACGCCG) relative to the translational start site (+1). These sites correspond to the region investigated in previous studies on adverse life experiences and related disorders including depression^21–24^.

Information on the features that could potentially affect cardiac outcomes^25^ was obtained. Demographic characteristics were collected on age, gender, education, marital status, living alone, housing, and employment status. For depression characteristics, the score from the self-completed Beck Depression Inventory (BDI)^26^ and previous and family histories of depression were recorded. For cardiac characteristics, ACS diagnosis (MI or UA), previous and family histories of ACS, diagnosed hypertension and diabetes mellitus, and reported current smoking status were ascertained. The following cardiovascular risk markers were measured: echocardiography for left ventricular ejection fraction (LVEF) and wall motion; electrocardiography for heart rate, PR interval, QRS duration, and QTc duration; measurements of body mass index (BMI) and blood pressure; and laboratory tests for troponin I, creatine kinase-MB (CK-MB), tumor necrosis factor-α, Interleukin (IL)-1β, IL-6, IL-18, high sensitivity C-reactive protein (hs-CRP), homocysteine, total- and lower density lipoprotein (LDL) cholesterol, and triglyceride levels.

### Long-term cardiac outcomes

Long-term cardiac outcomes were comprehensively evaluated using KAMIR data because detailed electronic data regarding hospital admissions, deaths, recurrent MI, and percutaneous coronary intervention (PCI) was stored in the KAMIR registry. All baseline participants in the present study were followed up for these outcomes. To implement non-hierarchic endpoint analyses, all patients were followed for the evaluation point of interest or until death. The primary endpoint was determined as a major adverse cardiac event (MACE), a defining composite of all-cause mortality, MI and PCI. Secondary endpoints were selected as all-cause mortality, cardiac death (determined as sudden death without other causes, death from arrhythmias or after MI or heart failure, or death caused by heart surgery or endocarditis), MI, and PCI. All potential events were adjudicated by an independent endpoint committee composed of study cardiologists who was blinded to participants’ depression comorbidity.

### Statistical analyses

Using the sample median value, *NR3C1* methylation percentages were classified by a binary variable (‘lower’ or ‘higher’ categories), in line with previous research^27^. Demographic and clinical characteristics of ACS patients with lower and higher methylation were compared applying t-tests or χ^2^ tests, as appropriate. Correlations between the methylation percentage and cardiovascular risk markers were examined by the Spearman correlation coefficients, after partial for BDI scores. Factors potentially associated with methylation status (p < 0.05) and other characteristics found to possess significant effects on MACE^25,28^ were considered as covariates in subsequent adjusted analyses. Kaplan–Meier curves were constructed, and the cumulative proportions of MACE in those with lower vs. higher *NR3C1F* methylation values were compared using the log-rank tests, and further stratified by the presence or not of depressive disorders at baseline. Time to the first composite and individual MACEs were subsequently compared using Cox proportional hazards models, after adjustment for the potential covariates described above, between the lower vs. higher methylation groups in total participants and then by their depressive disorder comorbidity status. Additional sensitivity analyses were carried out applying the methylation value as a continuous exposure variable (10 percent unit increase) and as tertials (lower, middle, and higher) to re-examine its effect beyond the binary categorical approach. Schoenfeld residuals tests were carried out to test proportional-hazards assumptions in all models. Two-tailed tests were used in all of analyses to determine significance at the 5% level and all statistical analyses were conducted with SPSS 21.0 and STATA 12.0 software.

## Results

### NR3C1 methylation status and baseline characteristics

Of 969 participants, 378 (39%) ACS patients experienced depressive disorders at the baseline. Median (interquartile rage) and mean (standard deviation) value of average *NR3C1* methylation percentages and three individual CpG sites are summarized in online Table S1. Since the individual three CpG sites of *NR3C1* methylation percentages were closely correlated (all Spearman’s rho > 0.7, p-value < 0.001), the results regarding average CpG values were robust and similar but less obvious for individual CpG sites. Therefore, the results for average CpG values were solely presented in subsequent analyses. In Table 1, baseline characteristics of ACS patients with lower and higher average *NR3C1* methylation values are compared. The mean (standard deviation) and range of lower methylation group were 12.2 (5.2) and 0–20.4% while those of higher group were 31.2 (9.1) and 20.4–66.0%. A higher average methylation value was only significantly associated with higher BDI scores. No significant difference was found between those who did and did not agree to provide blood samples in terms of any baseline characteristic (all p-values > 0.15).

**Table 1:** Baseline characteristics by NR3C1 average methylation.

	Lower methylation (N = 484)	Higher methylation (N = 485)	Statistical coefficient	P-value^a^
**Socio-demographic characteristics**				
Age, mean (SD) years	58.7 (11.2)	57.8 (11.0)	t = +1.012	0.312
Gender, N (%) female	349 (72.1)	351 (72.4)	χ^2^ = 0.008	0.927
Education, mean (SD) years	9.9 (4.7)	9.8 (4.6)	t = +0.447	0.655
Unmarried marital status, N (%)	68 (14.0)	73 (15.1)	χ^2^ = 0.196	0.658
Living alone, N (%)	41 (8.5)	51 (10.5)	χ^2^ = 1.178	0.278
Housing, N (%) rented	68 (14.0)	82 (16.9)	χ^2^ = 1.512	0.210
Currently unemployed, N (%)	179 (37.0)	189 (39.0)	χ^2^ = 0.405	0.524
**Depression characteristics**				
BDI, mean (SD) score	8.3 (8.0)	11.8 (8.9)	t = −6.483	**<0.001**
Previous depression, N (%)	15 (3.1)	19 (3.9)	χ^2^ = 0.479	0.489
Family history of depression, N (%)	15 (3.1)	8 (1.6)	χ^2^ = 2.197	0.138
**Cardiac characteristics**, N (%)				
ACS diagnosis				
Myocardial infarction	368 (76.0)	346 (71.3)	χ^2^ = 2.751	0.097
Unstable angina	116 (24.0)	139 (28.7)		
Previous ACS	17 (3.5)	22 (4.5)	χ^2^ = 0.657	0.418
Family history of ACS	14 (2.9)	17 (3.5)	χ^2^ = 0.294	0.588
Hypertension	224 (46.3)	234 (48.2)	χ^2^ = 0.376	0.540
Diabetes mellitus	90 (18.6	101 (20.8)	χ^2^ = 0.761	0.383
Current smoker	174 (36.0)	192 (39.6)	χ^2^ = 1.363	0.243

^a^p-values using t-tests or χ^2^ tests as appropriate.

*BDI*, Beck Depression Inventory; *ACS*, acute coronary syndrome.

### Correlations between NR3C1 methylation percentage and cardiovascular risk markers

*NR3C1* average methylation percentage was significantly correlated with longer QTc duration, higher BMI, and higher serum levels of troponin I and CK-MB after partial for BDI scores (Table 2). These characteristics were entered as covariates in the following adjusted analyses. In addition, age, ACS diagnosis, previous ACS, hypertension, diabetes, smoking, LVEF, and/or depressive disorders were also included as covariates because of known association with cardiac outcomes in previous studies^25,28^.

**Table 2:** Spearman’s correlations between NR3C1 average methylation and cardiovascular risk markers at baseline partial for Beck Depression Index scores (N = 969).

	rho	P-value
Left ventricular ejection fraction, mean (SD) %	−0.040	0.210
Wall motion, mean(SD) score	+0.051	0.110
Heart rate, mean (SD) beats/min	+0.057	0.079
PR interval, mean (SD) ms	+0.054	0.090
QRS duration, mean (SD) ms	+0.004	0.909
QTc duration, mean (SD) ms	+0.069	**0.036**
Body mass index, mean (SD) Kg/m^2^	+0.073	**0.025**
Systolic blood pressure, mean (SD) mmHg	−0.001	0.987
Diastolic blood pressure, mean (SD) mmHg	+0.015	0.650
Troponin I, mean(SD) mg/dL	+0.066	**0.041**
Creatine kinase-MB, mean(SD) mg/dL	+0.066	**0.040**
Tumor necrosis factor-a, mean(SD) pg/mL	+0.040	0.211
Interleukin-1b, mean(SD) pg/mL	+0.009	0.779
Interleukin-6, mean(SD) pg/mL	−0.009	0.770
Interleukin-18, mean(SD) pg/mL	+0.018	0.574
High sensitivity C-reactive protein, mean(SD) mg/dL	+0.050	0.123
Homocysteine, mean(SD) μmol/L	−0.002	0.959
Total cholesterol, mean(SD) mg/dL	+0.021	0.513
Lower density lipoprotein cholesterol, mean(SD) mg/dL	+0.010	0.774
Triglyceride, mean(SD) mg/dL	−0.007	0.845

### Effects of *NR3C1* methylation percentage on prognosis of ACS

**Figure 1:**
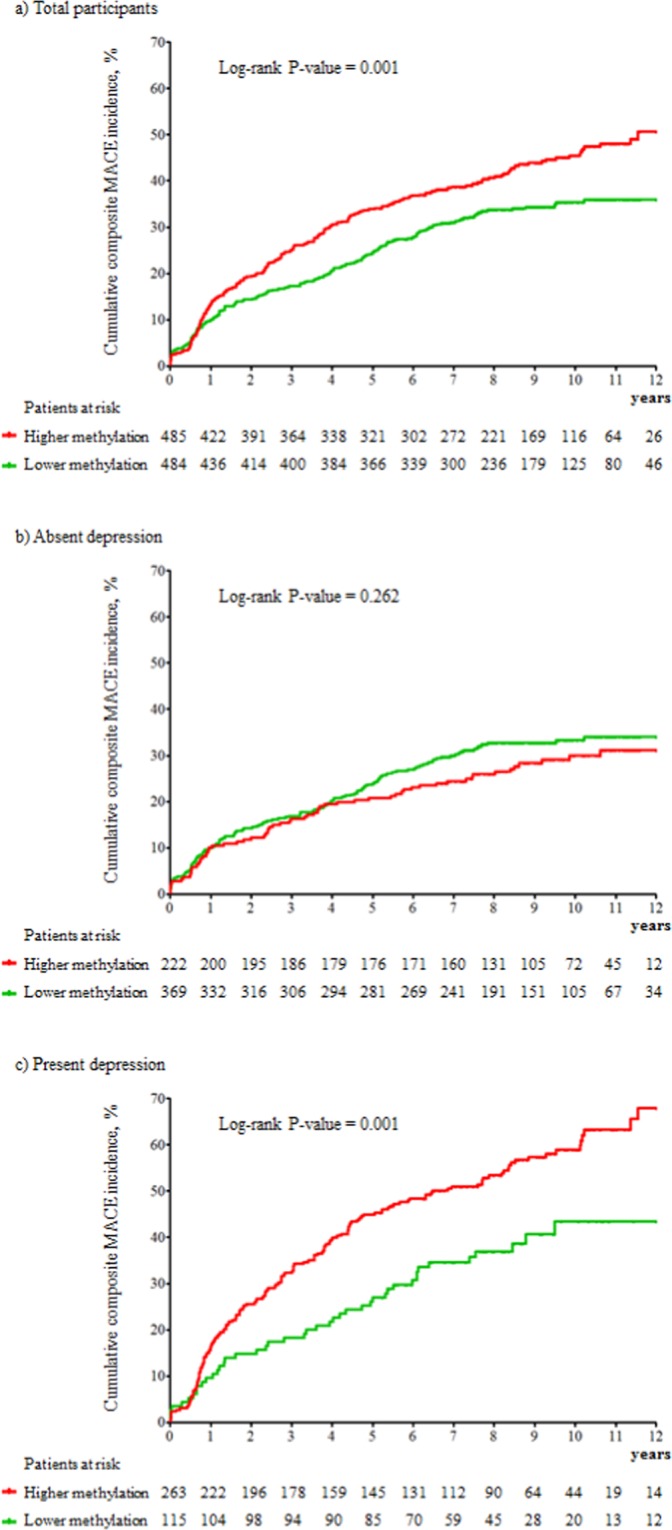
Cumulative incidence (%) of composite major adverse cardiac events (MACE) by NR3C1 average methylation and depressive disorder at baseline.

The cardiac outcomes of all participants were followed until 2017 or to their deaths [median; mean (standard deviation) duration of follow-up = 8.4; 8.7 (1.5) years]. 383 participants (39.5%) experienced the primary endpoint (composite MACE). Secondary endpoint numbers were as follows: 178 (18.4%) for all-cause mortality, 98 (10.1%) for cardiac death, 101 (10.4%) for MI and 139 (14.3%) for PCI. The cumulative risk of the composite MACE in subjects with lower and higher average *NR3C1* methylation is described in Fig. 1. A significant difference was found in the total sample. However, stratified by depressive disorder status, a significant group difference was found only in those with depressive disorder, and did not appear to be present in those without depressive disorder.

Comparisons of first MACE rates by lower vs. higher methylation values, successively adjusted for potential confounding variables, are summarized in Table 3. In the unadjusted analyses, a higher methylation was significantly associated with higher rates of composite MACE, all-cause mortality, MI, and PCI. The associations were weaker and remained significant only for composite MACE and MI outcomes after adjustment for age, ACS diagnosis, previous ACS, hypertension, diabetes, smoking, LVEF, QTc duration, BMI, and serum levels of troponin I and creatine kinase-MB. The strengths of the associations were substantially weaker after further adjustment for depressive disorder status, and the association was significant only for the composite MACE outcome.

**Table 3:** Associations of a higher NR3C1 average methylation at baseline with composite and individual components of major adverse cardiac events (MACE) in all patients with acute coronary syndrome (ACS).

	N (%) MACE		Hazard ratios (95% confidence intervals) [HRs (95% CIs)]		
	Lower methylation (N = 484)	Higher methylation (N = 485)	Unadusted	Adjusted^a^	Adjusted^b^
MACE	166 (34.3)	217 (44.7)	1.48 (1.20–1.81)^‡^	1.40 (1.14–1.71)^†^	1.25 (1.01–1.55)*
All-cause mortality	76 (15.7)	102 (21.0)	1.40 (1.04–1.89)*	1.35 (0.99–1.81)	1.24 (0.90–1.70)
Cardiac death	42 (8.7)	56 (11.5)	1.40 (0.94–2.11)	1.34 (0.90–1.99)	1.23 (0.80–1.88)
Myocardial infarction	41 (8.5)	60 (12.4)	1.51 (1.01–2.26)*	1.49 (1.99–2.19)^*^	1.17 (0.77–1.78)
Percutaneous coronary intervention	60 (12.4)	79 (16.3)	1.45 (1.03–2.04)*	1.38 (0.99–1.93)	1.24 (0.87–1.77)

^a^Model 1: adjusted for age, ACS diagnosis, previous ACS, hypertension, diabetes, smoking, left ventricular ejection fraction, QTc duration, body mass index, and serum levels of troponin I and creatine kinase-MB.

^b^Model 2: additionally adjusted for depressive disorder status.

*p-value < 0.05; ^†^p-value < 0.01; ^‡^p-value < 0.001.

Comparisons of first MACE rates by lower vs. higher methylation values stratified by baseline depressive disorder after adjustment are summarized in Table 4. Higher methylation was significantly associated with higher rates of composite MACE, all-cause mortality, cardiac death, and PCI only in those with depressive disorder after adjustment for age, ACS diagnosis, previous ACS, hypertension, diabetes, smoking, LVEF, QTc duration, BMI, and serum levels of troponin I and creatine kinase-MB. The *NR3C1* methylation x depressive disorder multiplicative interaction terms were statistically significant for the composite MACE, all-cause mortality, and cardiac death outcomes. Results of additional sensitivity analysis using the methylation percentages as a continuous variable and as tertials (lower, middle, and higher) are displayed in online Table S2 and online Table S3 respectively: in summary, the strengths of the associations were not substantially changed using continuous variables while the strength of the associations were generally lost but the significance of the association with MACE was remained in those with depressive disorder after adjustment. Model assumptions were all met (Schoenfeld p-values > 0.30).

**Table 4:** Associations of a higher NR3C1 average methylation at baseline with composite and individual components of major adverse cardiac events (MACE) in patients with acute coronary syndrome (ACS) by depression depressive disorder status.

	Absent depressive disorder (N = 591)			Present depressive disorder (N = 378)			P-value for interaction
	N (%) MACE		HRs (95% CIs)	N (%) MACE		HRs (95% CIs)	
	Lower methylation(N = 369)	Higher methylation (N = 222)		Lower methylation (N = 115)	Higher methylation (N = 263)		
Composite MACE	122 (33.1)	64 (28.8)	0.97 (0.83–1.13)	44 (38.3)	153 (58.2)	1.98 (1.39–2.81)^‡^	0.001
All-cause mortality	58 (15.7)	29 (13.1)	0.95 (0.76–1.20)	18 (15.7)	73 (27.8)	1.90 (1.12–3.22)*	0.013
Cardiac death	32 (8.7)	14 (6.3)	0.89 (0.64–1.22)	10 (8.7)	42 (16.0)	2.24 (1.09–4.59)*	0.036
Myocardial infarction	29 (7.9)	16 (7.2)	0.93 (0.69–1.27)	12 (10.4)	44 (16.7)	1.78 (0.92–3.46)	0.177
Percutaneous coronary intervention	43 (11.7)	23 (10.4)	0.95 (0.73–1.22)	17 (14.8)	56 (21.3)	1.90 (1.09–3.32)*	0.157

Hazard ratio (95% confidence interval) [HR (95% CI)] was estimated with adjustment for age, ACS diagnosis, previous ACS, hypertension, diabetes, smoking, left ventricular ejection fraction, QTc duration, body mass index, and serum levels of troponin I and creatine kinase-MB at baseline.

*p-value < 0.05; ^†^p-value < 0.01; ^‡^p-value < 0.001.

## Discussion

The principal findings from this cohort study were that higher *NR3C1* methylation status was correlated with several cardiovascular risk markers at the early phase of ACS and predicted worse long-term prognosis. The longitudinal associations were significant only in the presence of depressive disorder with significant synergistic interaction terms, and were independent of a range of potential confounding factors.

In these patients with ACS, a higher *NR3C1* methylation value was significantly correlated with worse profiles of several cardiovascular risk markers including QTc duration, BMI, and serum troponin I and CK-MB. These findings are in line with recent studies reporting that hypermethylation of *NR3C1* is associated with atherosclerosis^11^, and with heightened cardiovascular reactivity^12^. Although functional levels including GR mRNA or cortisol levels were not measured in the present study, hypermethylation of the *NR3C1* has been found to be related with reduced GR mRNA level^8^ and abnormal cortisol response^22^, which might affect glucocorticoid receptor sensitivities in turn cause endothelial dysfunctions, atherosclerosis and impaired metabolic signaling^29,30^. Based on these, it can be postulated that the association between *NR3C1* hypermethylation and cardiovascular risk markers may underlie its deleterious effects on long-term prognosis of ACS. However, in the present study, the longitudinal associations were explained partially but not fully by these variables.

These prognostic effects were more strongly explained by depressive disorder status, in that they were significant only in the presence of depressive disorder, independent of cardiovascular risk markers. Several explanations are possible for the observed synergistic effects. With respect to biological mechanisms, *NR3C1* hypermethylation is associated with adverse profiles of cardiovascular risk markers as stated above. Added to this, depression itself is associated with alterations in pro-inflammatory cytokines, and with autonomic and platelet dysfunction, which also have adverse effects on cardiac outcomes^31,32^; the synergy may therefore reflect multiple risk pathway involvement. Considering behavioural aspects, *NR3C1* methylation has functional influences affecting GR expression and finally dysregulating stress responses via HPA axis^7–9^, which may result in a reduced ability to cope with stressful situations such as unexpected ACS itself, financial and occupational problems, disabilities and other difficulties faced by patients with ACS. That is, ACS patients with *NR3C1* hyper methylation, which was associated with cardiovascular risk markers, are more likely to also have depression. Moreover, depression has been associated with unhealthy behaviors such as sedentary lifestyle, and irregular hospital visits and medication taking, which themselves have negative effects on cardiac outcomes^33^. Summing up, ACS patients with both *NR3C1* hypermethylation and depressive disorder may be liable to poor prognosis through a combination of biological and behavioural factors.

As the first evaluation of these questions, this study has several strengths. Participants were consecutively recruited from all eligible patients with a recent ACS, which in turn contributed to decrease the risk of error originating from heterogeneous examination times and to increase the sample homogeneity. A depressive disorder was determined with a structured diagnostic interview, and psychiatric and cardiovascular characteristics were ascertained using well validated measurements. Vast amounts of covariates potentially affecting cardiac outcomes as well as methylation status were included in the present analyses. A single site recruitment was performed, which potentially results in a limitation of generalization of the present findings but possesses advantages in terms of consistency of evaluation and treatment. One important limitation of the present study is that only one CpG island in the *NR3C1* gene was examined, although this area has been repeatedly investigated in previous epigenetic studies pertaining to both depression and adverse life events^22,24,34^. Since this region was evaluated in relation to cardiac outcomes for the first time in our study, replication is needed. Attrition in the recruitment process can be another source of limitation because methylation analysis was available only in 84% of the baseline samples. However, no difference in the baseline demographic and clinical characteristics was found between patients with or without methylation analyses. Furthermore, long-term follow-up data on MACE have been completely collected for the primary analysis. Finial limitation was lack of functional measurement such as GR mRNA or cortisol levels. Although previous studies suggested that hypermethylation of NR3C1 methylation was associated with lower GR mRNA as well as abnormal cortisol response^8,22^, further investigations for underlying biological mechanisms between NR3C1 methylation and cardiac outcomes with interaction of depression.

In conclusion, higher *NR3C1* methylation status at early phase of ACS predicted worse long-term prognosis of ACS in the presence of depressive disorder, independent of potential confounders including important cardiovascular risk markers. Similarly, associations of depression with worse long-term cardiac outcomes were stronger in ACS patients with higher *NR3C1* methylation status. *NR3C1* methylation tests might have clinical implications in screening for epigenetic susceptibility to identify high risk groups of poor ACS prognosis; therefore, this status serves as a promising prognostic biomarker in these patients particularly with the diagnosis of depressive disorders. Future studies investigating the effects of drugs capable of regulating *NR3C1* methylation on prognosis of ACS are anticipated, and the present study may serve as a foundation for future research.

## Supplementary information

Supplementary information.
