# Supplementary material for: Synergistic effects of depression and NR3C1 methylation on prognosis of acute coronary syndrome
Source: Sci Rep. 2020 Mar 26;10:5519. doi: 10.1038/s41598-020-62449-2 (PMC7099028; doi:10.1038/s41598-020-62449-2)
Supplement: Supplementary file 1 — Supplementary information. [file 41598_2020_62449_MOESM1_ESM.docx]

**Synergistic effects of depression and *NR3C1* methylation on prognosis of acute coronary syndrome**

Hee-Ju Kang^1^, Robert Stewart^2,3^, Ju-Wan Kim^1^, Sung-Wan Kim^1^, Il-Seon Shin^1^, Min-Chul Kim^4^, Young Joon Hong^4^, Youngkeun Ahn^4^, Myung-Geun Shin^5^, Myung Ho Jeong^4^, Jin-Sang Yoon^1^, Jae-Min Kim^1^

***Supplementary information***

**SUPPLEMENTARY METHODS…………………………………………………………..2**

Eligibility Criteria for the DEPACS participants………………..…….……………………..2

NR3C1 methylation ………………..…….…………………………………………………3

**SUPPLEMENTARY FIGURE S1….……..………………………………………………..5**

**SUPPLEMENTARY FIGURE S2.…………………………………….….………………..6**

**SUPPLEMENTARY TABLE S1….……..…………………… ………….………………..7**

**SUPPLEMENTARY TABLE S2.……………….…………………... ……………………..8**

**SUPPLEMENTARY TABLE S3.……………….…………………... ……………………..9**

**Supplementary methods**

**Eligibility Criteria for the DEPACS participants**

For the DEPACS study entry, inclusion criteria were as follows: i) aged 18~85 years; ii) confirmed ACS by investigation (the presence of ST-segment elevation MI was determined by >30 min of continuous chest pain, a new ST-segment elevation ≥2 mm on at least two contiguous electrocardiographic leads, and creatine kinase-MB more than three times normal; the presence of non-ST-segment elevation MI was diagnosed by chest pain and a positive cardiac biochemical marker without new ST-segment elevation; and the presence of unstable angina was determined by chest pain within the preceding 72 h with or without ST-T wave changes or positive cardiac biochemical markers); iii) ability to complete study questionnaires; iv) ability to understand the study objectives and sign informed consent. Exclusion criteria were: i) occurrence of ACS while hospitalized for another reason; ii) ACS developing less than 3 months after a coronary artery bypass graft procedure; iii) uncontrolled hypertension (systolic blood pressure (BP) >180mmHg or diastolic BP >100mmHg); iv) resting heart rate <40/min; v) severe physical illnesses threatening life or interfering with the recovery from ACS; vi) persistent clinically significant laboratory abnormalities in complete blood cell counts, thyroid tests, renal function tests, and liver function tests.

***NR3C1 1F* methylation**

Genomic DNA (1 µg) was extracted from whole blood and blood coagulants were treated using the QIAamp DNA Blood Mini Kit (Qiagen, Valencia, CA, USA). The bisulfite treatment was then performed with DNA using the EpiTech Bisulfite Kit (Qiagen) following the manufacturer’s protocol. Amplification of a 406-bp fragment of the *NR3C1* gene was underwent by polymerase chain reaction (PCR) from bisulfite-treated DNA with the forward and reverse primers described in Figure 2. The PCR was conducted as following conditions; 94ºC for 15 minutes, followed by 45 cycles of 94ºC for 30 seconds, 58ºC for 30 seconds, and 72ºC for 40 seconds, with a final extension of 10 minutes at 72ºC. The PSQ 96M Pyrosequencing System (Biotage) was used to sequence the PCR products by identifying the nucleotide incorporated by a DNA polymerase. Pyro Q-CpG software, version 1.0.9 (Biotage) was used to quantify the methylation percentage at each CpG sites.

Three CpG sites in exon *1F* (Supplementary Fig. 2), which correspond to CpG 1-3 in the study by Perroud et al.^1^, CpG 40-42 in the study by Palma-Gudiel et al.^2^, and CpG sites 45-47 in the study by Daskalakis and Yehuda^3^ were selected based on their prominence in previous studies of adverse life experiences and related disorders, including depression^1,4-6^. In the present analyses, the individual methylation percentages at the three CpG sites and their average values were utilized.

**Reference**

1. Perroud, N. et al. Increased methylation of glucocorticoid receptor gene (NR3C1) in adults with a history of childhood maltreatment: a link with the severity and type of trauma. *Transl. Psychiatry* **1**, e59 (2011).
2. Palma-Gudiel, H., Córdova-Palomera, A., Leza, J.C. & Fañanás, L. Glucocorticoid receptor gene (NR3C1) methylation processes as mediators of early adversity in stress related disorders causality: A critical review. *Neurosci. Biobehav. Rev.* **55**, 520-535 (2015).
3. Daskalakis, N.P. & Yehuda, R. Site-specific methylation changes in the glucocorticoid receptor exon 1F promoter in relation to life adversity: systematic review of contributing factors. *Front. Neurosci.* **8**, 369 (2014).
4. Perroud, N. et al. The Tutsi genocide and transgenerational transmission of maternal stress: epigenetics and biology of the HPA axis. *World J. Biol. Psychiatry* **15**, 334–345 (2014).
5. Martin-Blanco, A. et al. Association between methylation of the glucocorticoid receptor gene, childhood maltreatment, and clinical severity in borderline personality disorder. *J. Psychiatr. Res.* **57**, 34-40 (2014).
6. Bustamante, A.C. et al. Glucocorticoid receptor DNA methylation, childhood maltreatment and major depression. *J. Affect. Disord.* **206**,181-188 (2016).

**Figure S1.** Flow diagram for the recruitment process.

ACS, acute coronary syndrome; BDI, Beck Depression Inventory; MINI, Mini-International Neuropsychiatric Interview.


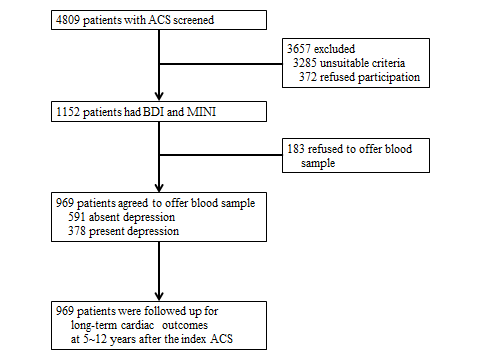
 **Figure S2.** *NR3C1* gene for analyzing methylation status

Figure legends

The CpGs are underlined and numbered. Forward and backward primers and sequencers are designated. The numbering of the gene sequence is relative to the translational start site (+1), which is located 13 nucleotides from the start of exon 2. Nucleotides given in lower-case letters represent intronic regions, whereas those in upper-case letters represent exon 1F.

The three CpG sites 1, 2, and 3 are similar to CpG sites 1, 2, 3 described by Perroud et al. (2011), and sites 45, 46, and 47 described by Daskalakis and Yehuda (2014).

| **Table S1 Descriptive data on NR3C1 methylation percentages at baseline (N=969).** | | |
| --- | --- | --- |
|  | Median (interquartile range) | Mean (standard deviation) |
| CpG average | 20.4 (12.4-28.4) | 21.7 (12.0) |
| CpG1 | 15.9 (9.9-23.7) | 17.3 (9.9) |
| CpG2 | 24.3 (14.0-34.0) | 25.2 (15.1) |
| CpG3 | 23.0 (11.0-30.0) | 22.7 (13.5) |

| **Table S2** **Associations of NR3C1 average methylation (per 10% unit increase) at baseline with long-term cardiac outcomes in patients with acute coronary syndrome (ACS) by depression depressive disorder status. Data are hazard ratios (95% confidence intervals)** | | |
| --- | --- | --- |
|  | Absent depressive disorder (N=591) | Present depressive disorder (N=378) |
| Major adverse cardiac events | 0.97 (0.92-1.02) | 1.11 (1.08-1.17)^‡^ |
| All-cause mortality | 0.98 (0.91-1.06) | 1.08 (1.02-1.16)^*^ |
| Cardiac death | 0.98 (0.88-1.01) | 1.11 (1.02-1.20)^†^ |
| Myocardial infarction | 0.94 (0.84-1.05) | 1.07 (1.00-1.14)^*^ |
| Percutaneous coronary intervention | 0.96 (0.88-1.05) | 1.12 (1.02-1.22)^*^ |
| Data were estimated adjusted for age, Beck Depression Inventory scores, ACS diagnosis, previous ACS, hypertension, diabetes, smoking, QTc duration, left ventricular ejection fraction, body mass index, and serum levels of troponin I and creatine kinase-MB at baseline.  ^*^p-value<0.05; ^†^p-value<0.01; ^‡^p-value<0.001. | | |

| **Table S3** **Associations of NR3C1 average methylation (stratified by tertials) at baseline with long-term cardiac outcomes in patients with acute coronary syndrome (ACS) by depression depressive disorder status. Data are hazard ratios (95% confidence intervals)** | | | |
| --- | --- | --- | --- |
| Outcomes | NR3C1 average methylation | Absent depressive disorder (N=591) | Present depressive disorder (N=378) |
| Major adverse  cardiac events | Lower | Ref | Ref |
|  | Middle | 1.00 (0.71-1.39) | 0.88 (0.55-1.41) |
|  | Higher | 0.73 (0.48-1.11) | 1.48 (1.02-2.13)^*^ |
| All-cause mortality | Lower | Ref | Ref |
|  | Middle | 0.97(0.59-1.60) | 1.11 (0.55-2.26) |
|  | Higher | 0.86 (0.47-1.55) | 1.58 (0.91-2.76) |
| Cardiac death | Lower | Ref | Ref |
|  | Middle | 1.45 (0.73-2.88) | 1.24 (0.46-3.35) |
|  | Higher | 0.74 (0.30-1.85) | 1.75 (0.81-3.78) |
| Myocardial infarction | Lower | Ref | Ref |
|  | Middle | 1.04(0.54-1.97) | 1.39 (0.55-3.51) |
|  | Higher | 0.50 (0.20-1.25) | 1.78 (0.84-3.79) |
| Percutaneous coronary intervention | Lower | Ref | Ref |
|  | Middle | 0.90 (0.52-1.58) | 0.71 (0.33-1.53) |
|  | Higher | 0.61 (0.30-1.27) | 1.53 (0.84-2.78) |
| Data were estimated adjusted for age, Beck Depression Inventory scores, ACS diagnosis, previous ACS, hypertension, diabetes, smoking, QTc duration, left ventricular ejection fraction, body mass index, and serum levels of troponin I and creatine kinase-MB at baseline.  ^*^p-value<0.05; ^†^p-value<0.01; ^‡^p-value<0.001. | | | |
